# Driver Identification System Using Normalized Electrocardiogram Based on Adaptive Threshold Filter for Intelligent Vehicles

**DOI:** 10.3390/s21010202

**Published:** 2020-12-30

**Authors:** Gyu Ho Choi, Kiho Lim, Sung Bum Pan

**Affiliations:** 1 IT Research Institute, Chosun University, Gwangju 61452, Korea; ghchoi@chosun.kr; 2 Department of Computer Science, William Paterson University of New Jersey, Wayne, NJ 07470, USA

**Keywords:** biometrics, driver identification, ECG, normalization, adaptive threshold filter, intelligent vehicle

## Abstract

Driver-centered infotainment and telematics services are provided for intelligent vehicles that improve driver convenience. Driver-centered services are performed after identification, and a biometrics system using bio-signals is applied. The electrocardiogram (ECG) signal acquired in the driving environment needs to be normalized because the intensity of noise is strong because the driver’s motion artifact is included. Existing time, frequency, and phase normalization methods have a problem of distorting P, QRS Complexes, and T waves, which are morphological features of an ECG, or normalizing to signals containing noise. In this paper, we propose an adaptive threshold filter-based driver identification system to solve the problem of distortion of the ECG morphological features when normalized and the motion artifact noise of the ECG that causes the identification performance deterioration in the driving environment. The experimental results show that the proposed method improved the average similarity compared to the results without normalization. The identification performance was also improved compared to the results before normalization.

## 1. Introduction

Smart cars with biometrics technology are recently evolving to identify drivers and provide services in the vehicle environment [1,2]. Smart cars are being studied and developed from control and communication technologies to driver-centered technologies that can provide driver security and services [3,4]. In order to identify a driver from outside the vehicle, initially, it was attempted to open the door of the vehicle and start the vehicle with physical security systems based on ownership. Later, it was developed into a technology with simple personal customized security systems using the driver’s bio-information that does not risk loss [5]. Like driver security technology, personalized services are provided by driver recognition technology using the driver’s bio-information inside the vehicle [6]. Once a driver is identified, the doors open, and the seats, mirrors, air conditioning, and audio are automatically adjusted to the settings optimized for the specific individual. Moreover, various entertainments can be provided as personalized services based on the driver’s mood and emotion, health status, stress and fatigue, and transmitting signals to each controller according to the situations [7,8].

Various user recognition technologies have been applied to provide driver-customized services in a smart car environment. Typical recognition technologies applied in smart cars include face recognition, motion recognition, state recognition, voice recognition, and driver identification. The facial recognition technology [9] determines the driver’s fatigue and mood in real-time using the in-vehicle cameras or various sensors to recommend a music list that suits the driver’s condition and taste. Motion recognition technology [10] recognizes the driver’s motion using cameras and various sensors in the vehicle and provides a convenient user interface (UI). The state recognition technology [11] recognizes the driver’s emotions and health conditions using the driver’s bio-information acquired from various sensors. Voice recognition [12] analyzes the driver’s voice and helps conveniently access a variety of entertainment. Driver identification technology [13] helps to perform driving tasks when a driver using the acquired bio-information in a vehicle is identified.

Among the recognition technologies, driver identification technology is the first required step to provide driver-customized services. This is a crucial step for driver security because it is linked to a driver-centered platform. A biometrics system using bio-information for driver identification is a technology that registers individual physical and behavioral features and recognizes them in real-time [14]. Physical characteristics include external information of the human body, such as fingerprint, face, and iris. Behavioral characteristics include external signals of the human body, such as voice, gait, handwriting, and internal signals of the body such as an electrocardiogram (ECG), electromyography (EMG), an electroencephalogram (EEG). It has been a known issue that a user identification system using external signals has a higher error rate with lower identification accuracy compared with a user identification system using bio-information [15]. A bio-information identification system based on physical characteristics acquired from the outside and surface of the human body has been analyzed with a high accuracy, but it has been internationally issued due to forgery and alteration cases and accidents [16,17]. Therefore, various identification systems using the bio-signals inside body are being researched and developed [18,19,20].

Among the bio-signals generated by electrical signals inside the body, a typical ECG has individual characteristics depending on the heart’s electrophysiological factors, the location, size, and physical condition of the heart. However, since it occurs as an electrical signal, the individual’s ECG signal is measured differently depending on the measurement environment as a behavioral characteristic [21]. In particular, the ECG signal acquired in the driving environment contains high motion artifact noise according to the complex state of the driver. Driver identification and recognition performance using an ECG including motion artifact noise is analyzed with a high error rate and low accuracy. In order to solve this problem, existing normalization methods consider only the static state and have a problem of distorting P, QRS Complexes, and T waves, which are morphological features including unique bio-information in ECG one cycle. In this paper, we propose an adaptive threshold filter-based driver identification system to solve the problem of distortion of the ECG morphological features when normalized and the motion artifact noise of the ECG that causes the identification performance deterioration in the driving environment. The existing normalization methods performed to match morphological features in the time, frequency, and phase domains, respectively, and converts the ECG cycles in a static state. The proposed normalization method improved the average similarity to 1.8%, 0.13%, and 2.1% by the Euclidean distance, Mahalanobis distance, and cosine similarity than before normalization. The identification performance using the proposed normalization method improved by 2.48% on average compared to the results without normalization, 0.82% higher than the time method, 0.21% higher than the frequency method, and 1.85% higher than the phase method. The rest of the paper is organized as follows. Section 2 provides the background of our work and Section 3 presents our proposed a normalization method and driver identification system. Section 4 discusses the experimental methods, experiment results, and future research direction. Finally, we conclude in Section 5.

## 2. Biometrics Technique Using ECG Signal for Intelligent Vehicle

In automated industrialization, intelligent vehicle technology has been chosen as a suitable business model. Marczewshka et al. [22] defined and analyzed suitable business models according to each source, and noted that the potential and business model. The automotive industry should suggest intelligent technologies that can be replaced in the future. Further, Bar et al. [23] defined the ideology of Hamburg’s smart city. There is a focus on increasing investments related to solving environmental problems from citizenship. Despite the criticism of urban digitalization, related fields are heavily invested for smart city. Research is being conducted on ICT-related vehicle sharing systems & Intelligent vehicles for smart city. Eldijk et al. [24] quantified the effects of barriers to transport infrastructure. Travel time, selection, catchment, and service efficiency were quantified to measure direct traffic barrier effects. In particular, it must be developed as an intelligent vehicle in order to efficiently provide the vehicle service to the driver. Intelligent technologies installed in vehicles require artificial intelligence and biometrics systems for smart car environments. In particular, driver-centered infotainment and telematics services can be provided through biometrics in the vehicle. The biometrics system using bio-signals in vehicles is being installed. Biometrics research is underway in Internet of Things (IoT) environments and vehicle environments by utilizing an acquisition advantage of ECG signals as it can be easily measured with both hands. In IoT environments, a user identification technology using an ECG through a biometrics system has been developed to access a user-specific UI. In vehicle environments, a technology that a driver is identified using an ECG has developed to receive a driver-centered infotainment system.

### 2.1. Driver Status Recognition Technologies Using ECG Signal in Vehicle

**Figure 1:**
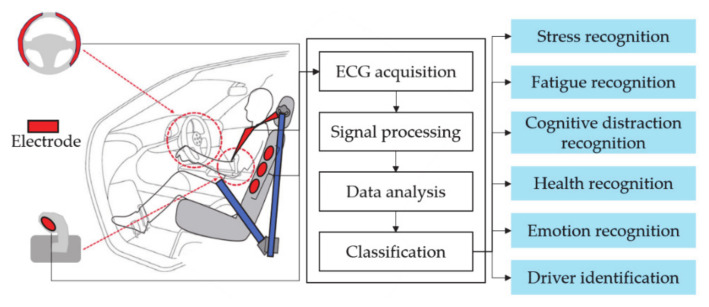
Driver identification and recognition technologies using ECG in vehicle environment.

In the biometrics system using an ECG, various recognition systems are applied in the vehicle environment to provide a driver-centered customized service as well as a user identification system in a real environment. Driver identification and recognition technologies using ECG in vehicle environment are described in Figure 1.

A recognition system using an ECG in a vehicle environment determines the driver’s condition and provides information through stress recognition, fatigue recognition, cognitive distraction recognition, health recognition, and emotion recognition. Nita et al. [25] proposed a system that can monitor the stress step by classification through morphological feature analysis and ECG acquisition from the chest at the seat belt position from the subject in a driving environment. Munla et al. [26] proposed a system that detects the driver’s stress level by measuring the ECG from the chest at the seat belt position in a driving environment and analyzing the heart rate variability (HRV). Wang et al. [27] proposed a driver’s fatigue recognition system by analyzing the R-R intervals of the ECG measured from the chest at the seat belt position in the driving environment. Shiwu et al. [28] proposed a driver fatigue recognition system by measuring ECG and EEG in a driving environment and analyzing various morphological feature data. Miyaji et al. [29,30] proposed a driver cognitive distraction recognition system that analyzes the RR interval of the ECG measured from the chest at the seat belt position, pupil diameter, gaze angle, and head rotation angle in the driving environment and notifies a warning to prevent vehicle accidents. Kumar et al. [31] proposed a system that monitors the driver’s health status by measuring multiple ECG from the driver’s seat and seat belt. Wang et al. [32] proposed an emotion recognition model using an ECG that measured driver’s calm and anxious emotions from the chest at the seat belt position for safe driving in a driving environment. Research on the driver’s identification and recognition system in the driving environment is underway, and the identification system must be conducted first in order to provide driver-customized services.

### 2.2. User Identification Using ECG Signal for Real Enviroment

**Figure 2:**
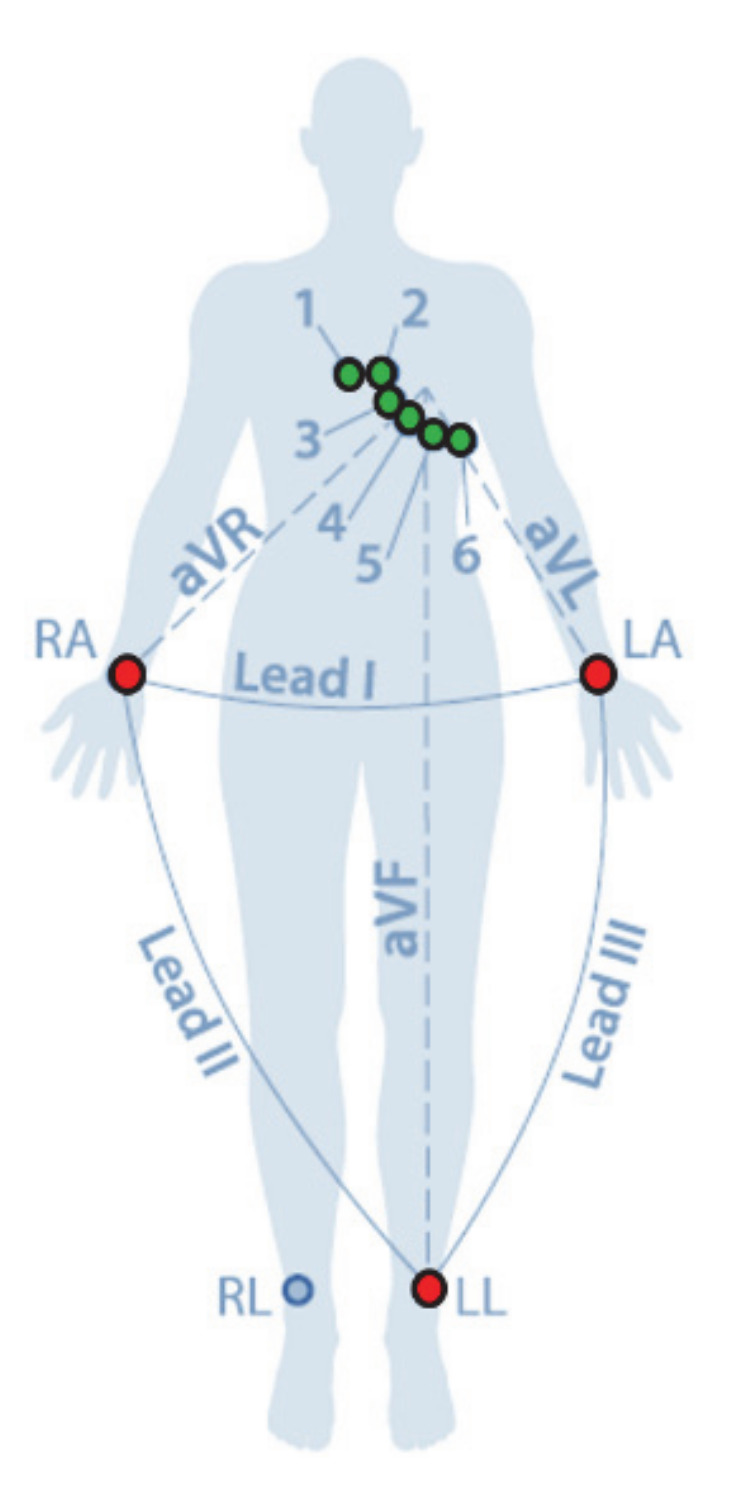
Medical acquisition settings: electrode placement and leads on the standard 12-lead configuration.

In the study of user identification using ECG in a real environment, a portable measuring device is used at a location where an ECG can be measured conveniently from users. ECG signals can be measured by standard lead as I, II, and III signals, limb lead as aVR, aVL, aVF signals and chest lead as V1, V2, V3, V4, V5, and V6 signals, respectively, as shown in Figure 2.

Since the ECG lead-I signal can be easily measured, the ECG lead-I signal is mainly used for user identification [33,34]. Table 1 shows each system and performance analyzed for user identification using an ECG in real environment.

Choi et al. [35] acquired the ECG Lead-I signal from 175 subjects in a static state from a mobile sensor and built a database (DB) consisting of ECG signals. The user identification performance was analyzed as 95.4% through the preprocessing process using a frequency filter, morphological feature extraction from one ECG cycle, and support vector machine (SVM). Eun et al. [36] acquired an ECG lead-I signal of 28 subjects in a static state and during slow walk through the developed measuring device that a watch-type wearable sensor, and built a DB. The user identification rate was 5.2% false acceptance rate (FAR), an indicator of authentication performance through the pre-processing process of normalization using frequency filter and Cross Correlation (CC) based on to check the similarity of two signals, morphological feature extraction in one ECG cycle, and Euclidean distance. S. Y. Chun [37] acquired the ECG Lead-I signal from 15 subjects in a static state from Nymi band, a watch-type wearable sensor, and constructed a DB. The preprocessing process using a frequency filter, the process of converting one ECG cycle into Short Time Fourier Transform (STFT) based on simultaneous analysis of time frequency components for features extraction, and the user identification rate was 2.2% Equal Error Rate (EER), an indicator of authentication rate by the Euclidean distance. Arteaga-Falconi et al. [38] acquired the ECG Lead-I signal from 73 subjects in a static state from a mobile sensor, and constructed a DB. The user identification accuracy was 84.93% through the preprocessing process using frequency filter and normalization, morphological feature extraction from one ECG period, and Euclidian distance. Sung et al. [39] acquired the ECG lead-I signal of 55 subjects from the measurement sensor MP-150 and constructed a DB. The user identification accuracy was 96.55% through the preprocessing step using a frequency filter, extraction of morphological features from one cycle of the first & second differential ECG signal, and reduction of the feature data dimension using Linear Discriminant Analysis (LDA). In consideration of the actual environment, a user identification system using an ECG has been analyzed with high performance and is being applied in the IoT field. Perio-Lopez et al. [40] defined non-legitimate users as an attacker to prevent attackers from accessing the user’s UI and proposed a continuous user identification system using ECG. Barros et al. [41] proposed an identification system that an ECG acquisition and 14 drivers identified to access objects through sensing in a driving environment. The user identification system considering the real environment has been studied to remove the noise of the ECG by considering only static and single states. This should remove ECG artifact noise acquired by the driver’s movement according to the complex state in the driving environment. The artifact noise of the ECG acquired by the driver’s movement in the driving environment degrades the performance of the driver identification system accuracy.

### 2.3. Driver Identification Using ECG Signal in Vehicle

In order to apply a driver-centered customized recognition system in a driving environment, the driver identification should be considered as a priority. In a vehicle, a driver identification system has been studied using not only the driver’s ECG, but also engine control unit (ECU), global positioning system (GPS), and impedance information. Rettore et al. [42] proposed a driver identification system that can contribute to driver assistance systems and intelligent traffic systems in Vehicular Ad-hoc Networks (VANET) based on car-to-car ad-hoc mobile communication and networking. The proposed driver identification system was analyzed with 98% performance using visual data from ECU. Rahim et al. [43] proposed a driver identification system with driving patterns using GPS information. It identifies the driver with 96% accuracy using features such as zero-to-stable elapsed time, direction change, stable speed, and overall acceleration. Since the driving pattern information is not unique to the driver, the driving pattern data can be duplicated and used maliciously. Roeschilin et al. [44] proposed a driver identification system using driver’s impedance. It measures the driver’s impedance from the vehicle’s steering wheel and identifies the driver with 92% accuracy. Impedance is the driver’s unique biometric information, but it has a disadvantage of being analyzed with low identification performance. Silva et al. [45] proposed a system that identifies the driver using the driver’s electrocardiogram in the driving environment. It measures the driver’s ECG from the vehicle’s steering wheel and identifies the driver with an error rate of 3 to 5% in a static state and a 30% error rate in a dynamic state. Santos et al. [46] proposed a driver identification system using the stress recognition in automobile driver’s DB provided by Physionet. This system identifies the driver with 95% accuracy by extracting features from the segmented ECG based on the fiducial point.

The ECG signal is an electrical signal generated by behavioral characteristics and the ECG acquired in the dynamic state of the driver by the driving environment must be normalized as shown in Table 2. Pinto et al. [47] proposed a driver identification system using a normalized ECG according to the driver’s condition. This system identifies the driver with 94% accuracy using features extracted from the ECG normalized by CC. Nobunaga et al. [48] proposed a normalization method that converts the ECG generated by the accelerated heart rate into an ECG signal when it is static. The ECG signal measured from the chest at the seat belt position is filtered in the optimal frequency band with the optimized band pass filter (OBPF) method. Lin et al. [49] proposed a normalization method that considers the state of the ECG, which is measured with noises included by movement. In the dynamic state, the ECG cycles measured from the metal rod electrodes are equally normalized in the phase domain. Although a study to normalize the ECG in a complex state is underway, if the morphological features are distorted or the input signal is composed of noise, a problem of normalization to a noise signal may occur. Considering the driving environment, the conventional normalization method has been studied in the time, frequency, and phase domains, but there is a problem of distorting the morphological features of the ECG. In this paper, we proposed a driver identification system based on a normalization method that filters ECG cycles by a driver-specific threshold to solve above issue.

## 3. Driver Identification System Using Adaptive Filter-Based Normalization Method

**Figure 3:**
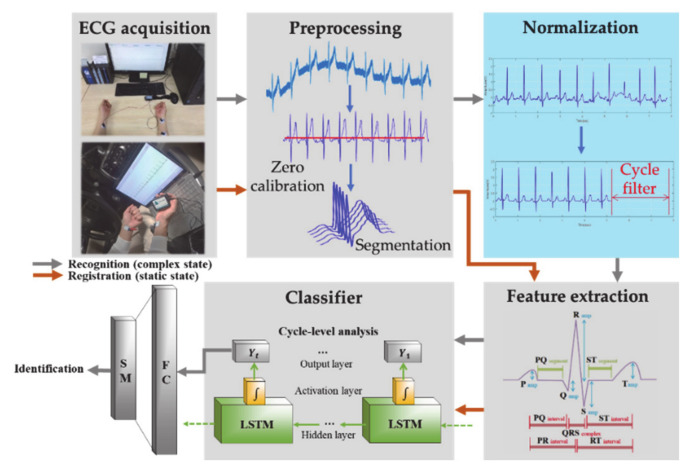
Driver identification and recognition technologies using ECG in vehicle environment.

The flowchart of the identification system to recognize the driver is shown in Figure 3. The system proposed to be installed in a vehicle mainly consists of a sensing unit which is a process of acquiring an ECG and an operation unit which processes the acquired ECG data. Therefore, a driver can be identified from a sensing element that precisely acquires ECG on the vehicle’s steering wheel, seat belt, gear rod, and hardware that can operate the acquired ECG data. If the vehicle is capable of acquiring an ECG and operating data without being interfered with by the vehicle’s electromagnetic signals, the proposed identification system with normalization method is applied to identify the driver. The driver identification system consists of a process of acquiring an ECG Lead-I signal, a preprocessing process to remove noise, a normalization process based on the proposed adaptive filter, and a process of identifying the driver from the classifier after feature extraction. The ECG acquired in a static state is stored as registration data. The stored data is modeled by learning by long short term memory (LSTM) based on learning technology that protects and controls long time series data. The recognition data are composed of ECG acquired in a complex state as well as a static state.

### 3.1. Driver Identification System

The driver identification system proceeds by applying the proposed adaptive filter-based normalization method and is divided into registration and recognition processes. The registration and recognition process are the same in the rest of the steps except for normalization until the feature extraction step, and the registration data is trained and modeled. The recognition data is calculated with the modeled registration data and classified. The registration data uses an ECG signal acquired in a static state. The recognition data uses an ECG signal acquired in a complex state according to driving environments. ECG signals were acquired with lead-I based on international standard 12 lead and built a DB. Noise included in the acquired ECG signal is removed through a frequency filtering process, an R-wave peak detection process, and a moving average filter process excluding the QRS Complexes section that contains unique personal information. The high-frequency component noise and the low-frequency component noise are removed by the Butterworth bandpass filter. The R-wave peak is detected from the threshold value by the Pan & Tomkins algorithm [50]. The R-wave peak detected by the algorithm is used as a fiducial point for setting the QRS Complexes section to apply the moving average filter. Even if noise is removed with frequency filtering and moving average filter, baseline wander noise caused by driver’s breath is not removed. To remove the baseline wander noise, a continuous first-order regression analysis method that minimizes morphological features in one cycle is applied and calibrated to zero [51]. The ECG signal adjusted to the zero points is segmented every cycle to constitute registration and recognition data. The ECG segmentation method is divided into a fiducial point and a non-fiducial point method. The fiducial point segmentation method using the morphological features of the ECG has been analyzed with higher identification performance than the non-fiducial point method [52]. In this paper, in order to divide a cycle from an ECG obtained by applying the fiducial point segmentation method, 0.3 sec to the P wave section to the left of the R wave peak detected by the Pan & Tomkins algorithm and 0.4 sec to the T wave section are set. The proposed adaptive filter-based normalization method filters through first-order from an ideal ECG and second-order filtered from an individual’s ideal ECG using selected one cycle. Feature extraction using a normalized ECG selects one cycle data of the ECG as morphological feature data. Classification for final driver identification is carried out by LSTM.

### 3.2. Normalization Based on Adaptive Filter

**Figure 4:**
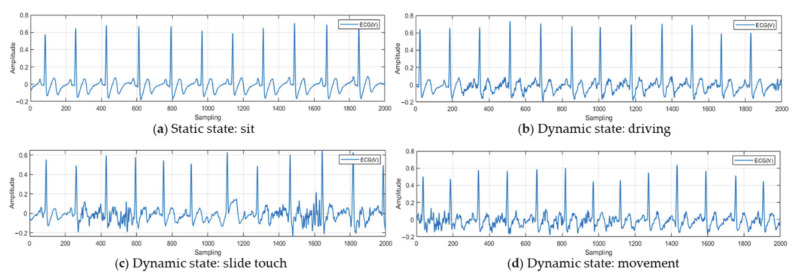
ECG acquired according to driver state: (**a**) static state: sit (**b**) dynamic state: driving (**c**) dynamic: slide touch (**d**) dynamic state: movement.

The ECG signal measured in a complex state may appear as a noise-like signal the artifact is mostly contained by movement as shown in Figure 4. In order to remove noise and match the morphological features of the ECG signal, the existing normalization method is an adaptive normalization method for ECG measured in a static state. Since registration data are not defined and there are too many cases until the recognition data are normalized and generated, it can be normalized to an ECG signal containing noise, which is problematic.

**Figure 5:**
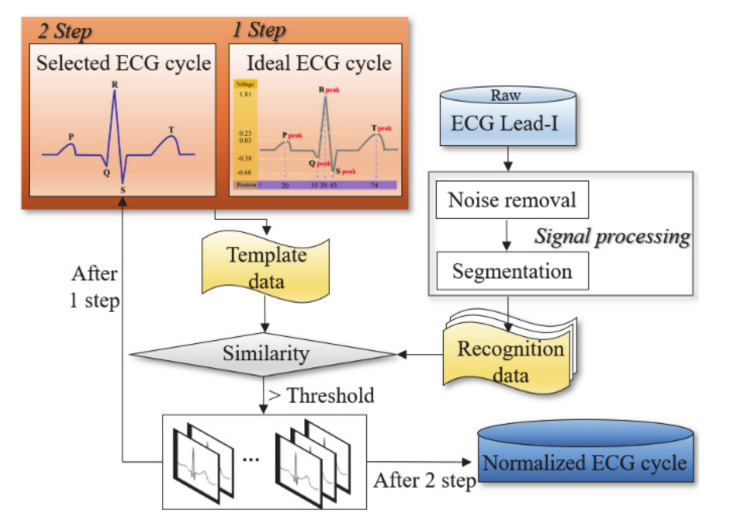
ECG normalization diagram.

In this paper, in order to normalize the morphological features of the ECG signal measured in the complex state to a distinct ECG cycle, we propose a method in which the ideal ECG cycle of each subject is selected, and the recognition data is filtered to the ideal ECG and then normalized. The proposed normalization method is composed of the process of generating an ideal ECG cycle, the process of selecting the adaptive sample ECG cycle of each subject, and the process of filtering the recognition data by the sample ECG cycle, as shown in Figure 5.

An ideal ECG cycle is a signal that consists of the P, QRS Complexes, and T waves of the ECG, and the signal is not crushed or distorted by noise. An ideal ECG one cycle is generated using the information of the peaks of P, Q, R, S, and T waves using the public database MIT-BIH Normal Sinus Rhythm (NSR) shown in Table 3, and the connection of the peaks is generated by interpolation based on a two-dimensional equation. PV, QV, RV, SV and TV is voltage of P, Q, R, S, T peak and PL, QL, RL, SL, and TL is the location of P, Q, R, S, and T peak in Table 3.

**Figure 6:**
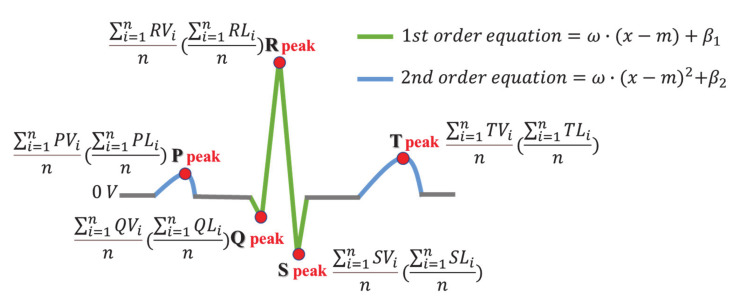
Generated ideal ECG one cycle.

The information on the peaks of P, Q, R, S, and T waves are voltages and data positions for the average of 100 cycles per subject of MIT-BIH NSR. The ideal ECG one cycle generated using the information on the average of the vertices is shown in Figure 6. 1st and 2nd order equations were used to interpolate the data excluding the peaks of each wave. Weight ω and bias β are calculated by Equation (1).

$$\omega =\frac{y_{2}-y_{1}}{x_{2}-x_{1}}$$

(1) $$\beta_{1}=\;y_{1}-\frac{y_{2}-y_{1}}{x_{2}-x_{1}}\cdot \left(x_{1}-n\right)$$

$$\beta_{2}=\;y_{1}-\frac{y_{2}-y_{1}}{x_{2}-x_{1}}\cdot {\left(x_{1}-n\right)}^{2}$$

**Figure 7:**
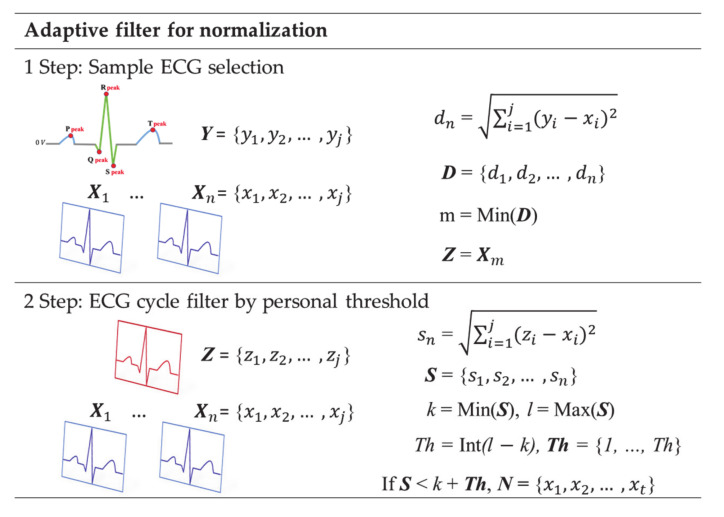
Adaptive filter process for normalization.

For each subject’s adaptive sample ECG, an ECG cycle in which the generated ideal ECG and the measured ECG correspond to the Euclidean distance’s Maximum Similarity Rate (MSR) are selected as the individual sample ECG. To select a sample ECG, the ECG must be measured in a static state in which the morphological features of the ECG signal can be clearly displayed. To analyze the similarity rate, the measured signals are divided into a cycle based on a fiducial point and matched to the same data size with the ideal ECG as shown in Figure 7, 1 step. *X* is the ECG cycle of the recognition data, *Y* is the ideal ECG cycle, and *Z* is the sample ECG cycle. When an individual’s sample ECG is selected, the recognition data can be normalized to the sample ECG as step 2. *S* is the similarity between the individual’s ideal cycle and the recognition data, and *N* is the cycles filtered by the threshold. The process of filtering the recognition data with the sample ECG is normalized by using the Euclidean distance and adjusting the individual’s threshold in the same way as the method of selecting the sample ECG.

**Figure 8:**
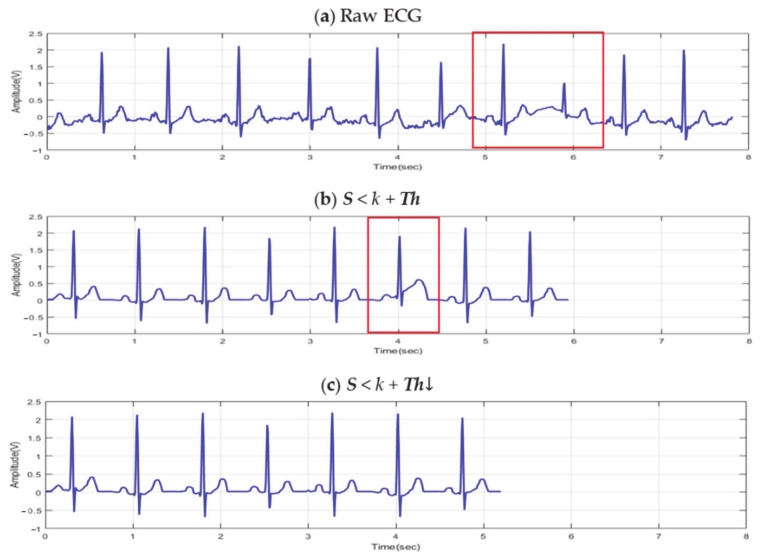
ECG cycle filtered as PSR decrease: (**a**) raw ECG (**b**) *S* < *k* + *Th* (**c**) *S* < *k* + *Th*↓.

The threshold value is composed of the number of integers between the maximum value *l* and the minimum value *k* of *S*. The higher the threshold value, the lower the number of ECG cycles filtered by the permit similarity rate (PSR), and the lower the threshold value, the number of filtered ECG cycles increases. Figure 8 shows that *Th* decreases from Figure 8b–c in the raw ECG signal. As the filtered ECG cycle increases, the ECG cycle consisting of individual morphological features is more similar.

**Figure 9:**
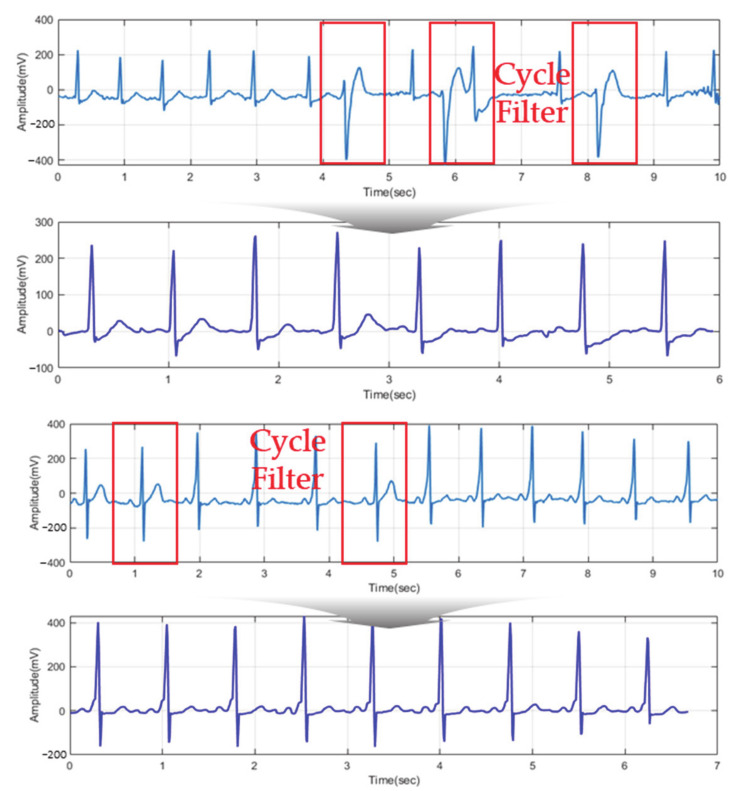
ECG signal normalized by adaptive filter for each subject.

Figure 9 shows ECG signals normalized according to the PSR values of each subject. If the morphological features are not identified as the same according to the similarity rate of the sample ECG of the individual from the ideal ECG, then they are filtered and excluded from the total signal. Therefore, by the proposed normalization algorithm, individual morphological features are normalized to similar ECG cycles and filtered.

## 4. Experiment Results

Experiments to analyze the proposed adaptive filter-based normalization method and the performance of the driver identification system were conducted in Matlab R2020b on a PC with an Intel Core i7 processor, NVIDA GeForce RTX 2060. The ECG signals for analyzing the performance were obtained from 100 subjects (64 males and 36 females) affiliated with Chosun University, and the age of the subjects ranged from 23 to 34 years old. The protocol for acquiring ECG signals is defined as shown in Table 4.

**Figure 10:**
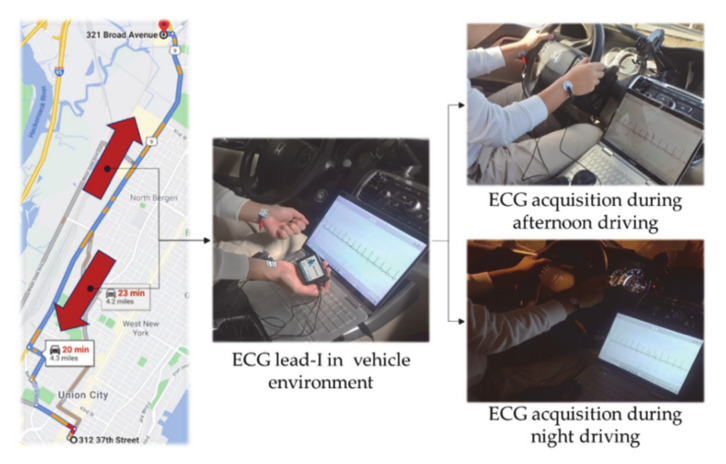
ECG lead-I acquisition in driving environment.

All subjects acquired a total of 3 times at intervals of 2–3 days. In consideration of the driving environment, it was performed in a seated state, a slide touch, and a post-exercise state for a fast heart rate. The ECG measurement equipment was BioPac’s MP-160, and Lead-I signals were acquired from both wrists using Corvidien’s wet electrode at a 2,000Hz sampling rate. ECG acquisition of the actual driving environment was carried out in the afternoon and at night while driving Tonnele Ave road from 312 37 St Union City NJ to 321 Broad Ave and Ridgefield NJ, as shown in Figure 10. The evaluation of the proposed normalization method using the measured ECG signals was compared and analyzed with the average similarity of ECG cycles and driver identification performance before and after normalization. The similarity analysis is performed according to each similarity evaluation method using the proposed normalization method and the existing time, frequency, and phase normalization methods. The similarity evaluation method is performed by the Euclidean distance, Mahalanobis distance, and cosine distance Equations (2)–(4), respectively.

(2) $$d\left(p,\;q\right)=\;\sqrt{\overset{n}{\underset{i=1}{\sum }}{\left(q_{i}-p_{i}\right)}^{2}}$$

(3) $$d\left(p,\;q\right)=\sqrt{\left(p-q\right)C^{-1}{\left(p-q\right)}^{T}}$$

(4) $$\cos\left(\theta \right)=\frac{p\cdot q}{\left\|p\|\|q\right\|}=\frac{\sum_{i=1}^{n}p_{i}\times q_{i}}{\sqrt{\sum_{i=1}^{n}p_{i}^{2}}\times \sqrt{\sum_{i=1}^{n}q_{i}^{2}}}$$

**Figure 11:**
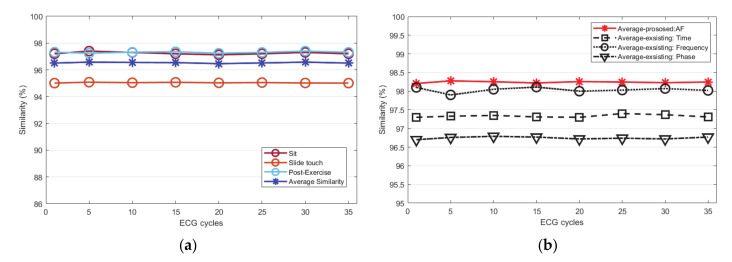
Euclidean distance similarity performance analysis: (**a**) similarity of each status before normalization (**b**) similarity after normalization (proposed method, existing method).

The Euclidean and Mahalanobis distances are similar as they get closer to zero by calculating the distances *p* and *q* of the two ECG cycle data. The closer the cosine distance is to 1, the more similar it is. In order to analyze not only the straight distance of the ECG cycles but also the distance of probability, the similarity of the ECG cycle is analyzed using the Mahalanobis distance. For the evaluation of similarity, the ECG cycle used for each subject was from 1 to 35. The number of similarities from 0 to 1 was inversely converted by the Euclidean distance and measured as a percentage. To analyze the similarity of the ECG, it was compared with the existing normalization method. The existing time normalization method is matched by a threshold value using a cross correlation algorithm. In the frequency normalization method, ECG signals are filtered by an optimal frequency band. In the phase normalization method, the ECG signal is mapped with a value delayed by τ as a phase trajectory. Figure 11a is a graph that averages the similarity performance according to the increase in the number of ECG cycles by the Euclidean distance before normalization. According to all conditions, the ECG cycle similarity performance was measured as an average for each number of cycles and totally analyzed as 96.5%.

**Figure 12:**
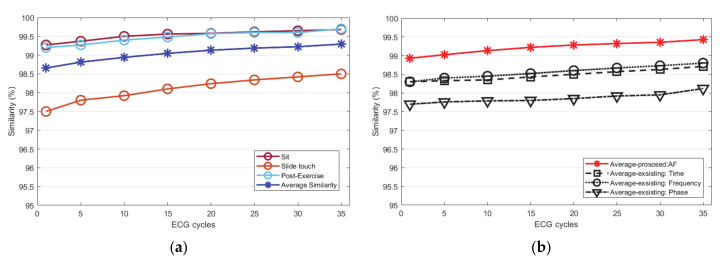
Mahalanobis distance similarity performance analysis: (**a**) similarity of each status before normalization (**b**) similarity after normalization (proposed method, existing method).

**Figure 13:**
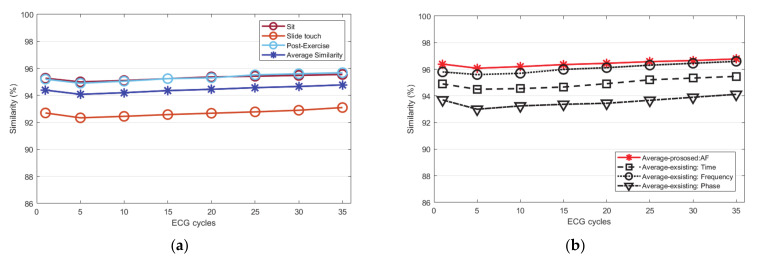
Cosine similarity performance analysis: (**a**) similarity of each status before normalization (**b**) similarity after normalization (proposed method, existing method).

Figure 11b compares and analyzes the average similarity performance according to the proposed normalization method and the existing normalization methods. The proposed method improved by an average 1.8% than before normalization. Among the existing methods, the frequency normalization method was analyzed as similarity rate to the proposed method, but it was an 0.25% lower on average. Other methods, including time and phase domain normalization, were analyzed lower than the frequency normalization method. Figure 12a is a graph that analyzed the similarity performance by the Mahalanobis distance before normalization as an average as the number of ECG cycles increased. This is the performance analyzed by the average similarity between a sitting state, a static state, driving state, post-exercise state. According to all conditions, the ECG cycle similarity performance was measured as an average for each number of cycles and analyzed as 99.2%. Figure 12b compares and analyzes the average similarity performance according to the proposed normalization method and the existing normalization method. The proposed method is improved by 0.13% on average compared to before normalization. Among the existing methods, the frequency normalization method was analyzed as similar to the proposed method, but the average was 0.62% lower. Other time and phase domain normalization methods were analyzed lower than that of the frequency normalization method. Figure 13a is a graph that analyzes the similarity performance as an average as the number of ECG cycles increases by cosine similarity before normalization. It is the performance analyzed by the average similarity between sitting state, static state, post-exercise state, and driving state. According to all conditions, the ECG cycle similarity performance was measured as an average for each number of cycles, and it was analyzed as 94.77%. Figure 13b compares and analyzes the average cosine similarity performance according to the proposed normalization method and the existing normalization method. The number of similarities from −1 to 1 was measured as a percentage by cosine similarity. The proposed method improved by 2.1% on average compared to before normalization. Among the existing methods, the frequency normalization method was analyzed as similar to the proposed method but was analyzed at an average of 0.17% lower. Other time and phase domain normalization methods were analyzed lower than the frequency method.

Driver identification performance analysis was measured by accuracy. The classification rate is a measure of the degree to which a class of subjects identified from all registered subject classes is predicted and matched. The classification rate measurement is calculated as Equation (4) by calculating the values of true positive (*TP*), true negative (*TN*), false positive (*FP*), and false negative (*FN*) predicted by 1:N matching.

(5) $$Accuracy=\frac{TP+TN}{TP+FN+FP+TN}$$

The ECG acquired in the static state was used as registration data, and the ECG acquired in the complex state was used as recognition data. As for the classification performance of the recognition data from the registration data, the recognition performance was analyzed as the ECG cycle increased through the classifier before normalization and using each normalization method. In this paper, the identification accuracy was analyzed using the LSTM, a deep learning technique, to classify the ECG in a complex state. LSTM is a machine learning technique that protects and controls long time series data by additional gates and cells in the recurrent neural network (RNN) structure that repeats and persists previous data. The hyperparameters of LSTM used in the experiment are shown in Table 5.

The structure of deep learning consists of 3 layers of LSTM, 2 fully connected layers (active function: ReLU), and 1 output layer (active function: softmax). In the case of the fully connected layer, a dropout is applied that ignores 50% of nodes in order to prevent overfitting in which the classification algorithm is adapted to registration data. For the optimization of each network, the learning rate rmsprop was 0.001, and the registration data were evaluated by the network adjusted with 10 epochs.

**Figure 14:**
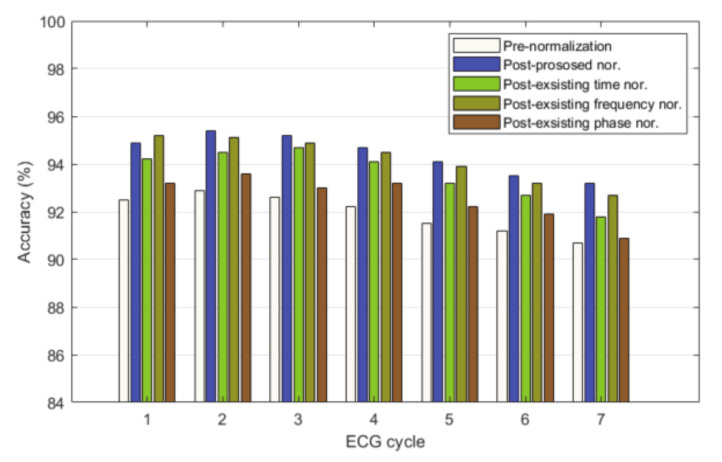
Comparison of identification performance using existing and proposed normalization.

Recognition performance was compared and analyzed using the proposed normalization method and the existing normalization method. The existing normalization method was performed with CC of time, OBPF of frequency, and phase. Figure 14 is a graph comparing and analyzing the recognition performance before normalization and as the number of ECG cycles increased by each normalization method. The identification performance using the existing normalization method increased by 1.66% (time) on average, 2.27% on average (frequency), and 0.63% (phase) on average compared to before normalization. The identification accuracy using the proposed normalization method increased by 2.48% on average compared to before normalization. The time normalization method was analyzed as 94.7% of the best identification performance in the ECG cycle 3, the frequency normalization method was 95.2% accuracy of the best identification performance in the ECG cycle 1, and the phase normalization method was analyzed as 93.6% of the best identification performance in the ECG cycle 2. The proposed adaptive filter normalization method was analyzed as 95.4% of the best identification performance in the ECG cycle 2 and higher identification performance was identified than existing normalization methods.

## 5. Conclusions

In the driving environment, an electrical signal acquired in the ECG contains a lot of noise due to the driver’s behavioral characteristics and cannot be used to identify the driver. The ECG signal acquired for driver identification is measured with different morphological features according to the measurement environment, and as artifacts are generated mostly by the driving environment, identification performance is degraded. Existing normalization methods have been carried out by distorting the morphological features without considering the driving conditions. In this paper, we proposed a driver identification system based on a normalization method that filters ECG cycles by driver-specific threshold to solve the problem of distortion of the ECG morphological features when normalized and the motion artifact noise of the ECG that causes the identification performance deterioration in the driving environment. Noise-type ECG signals that degrade driver identification performance are removed by the proposed adaptive threshold filter and normalized to ECG with distinct morphological features. The proposed normalization method consists of a process of generating an ideal ECG cycle, a process of selecting an adaptive sample ECG cycle for each subject, and a process of filtering the recognition data by the sample ECG cycle. The driver is identified by LSTM using a normalized ECG cycle.

The Euclidean distance, Mahalanobis distance, and cosine similarity were improved to 1.8%, 0.13%, and 2.1% on average by the proposed normalization method using ECG acquired from 100 subjects. In addition, the identification performance of the proposed normalization method was improved by 2.48% on average compared to before normalization, 0.82% higher than the time method, 0.21% higher than the frequency method, and 1.85% higher than the phase method. As the morphological features of the ECG were matched by the normalization method, the identification performance was improved and analyzed. In the future, we plan to acquire ECG from many subjects in a long-term driving environment for driver identification and to develop deep learning algorithms for a multi-biometrics system.

## Figures and Tables

**Table 1:** User identification system analysis and performance using ECG in real environment.

Device	Status, Signal	Feature Analysis	Performance	Ref.
Mobile sensor (CardioChip)	Static, ECG lead-I	Morphological features	Accuracy 95.4%	[35]
Wearable sensor (Arm Cortex)	Slow walking, ECG lead-I	CC	FAR 5.2%	[36]
Wearable sensor (Nymi band)	Static, ECG lead-I	STFT	EER 2.2%	[37]
Mobile sensor (AliveCore)	Static, ECG lead-I	Morphological features	Accuracy 84.93%	[38]
Sensor (MP-150)	Post-exercise, ECG lead-I	1st and 2nd derivation	Accuracy 96.55%	[39]

**Table 2:** Preprocessing: ECG normalization considering driving conditions.

ECG Acquisition Location	Status	Normalization	Performance	Reference
Steering wheel	Driving	Cross Correlation	94%	[47]
Chest	Post-exercising	Optimized Band Pass Filter	100%	[48]
Metal rod electrode	Exercising	Phase	87%	[49]

**Table 3:** Average information on P, Q, R, S, T peaks of MIT-BIH NSR DB.

Subjects	Average Voltage (Location)				
	P Peak	Q Peak	R Peak	S Peak	T Peak
18	$\frac{\sum_{i=1}^{n}PV_{i}}{n}$($\frac{\sum_{i=1}^{n}PL_{i}}{n}$)	$\frac{\sum_{i=1}^{n}QV_{i}}{n}$($\frac{\sum_{i=1}^{n}QL_{i}}{n}$)	$\frac{\sum_{i=1}^{n}RV_{i}}{n}$($\frac{\sum_{i=1}^{n}RL_{i}}{n}$)	$\frac{\sum_{i=1}^{n}SV_{i}}{n}$($\frac{\sum_{i=1}^{n}SL_{i}}{n}$)	$\frac{\sum_{i=1}^{n}TV_{i}}{n}$($\frac{\sum_{i=1}^{n}TL_{i}}{n}$)

**Table 4:** ECG acquisition protocol considering the driving environment.

Times	ECG Acquisition Environment		
	Sit	Slide Touch	Post-Exercise
**1**	60 sec	10 times	180 sec
2–3 days break			
**2**	60 sec	10 times	180 sec
2–3 days break			
**3**	60 sec	10 times	180 sec

**Table 5:** Hyperparameter of LSTM.

1-Layer	2-Layer	3-Layer
In put layer (m, n)		
LSTM(n)	LSTM(n)	LSTM(n)
-	LSTM(n/2)	LSTM(n/2)
-	-	LSTM(n/3)
Fully Connected Layer (400), Dropout = 0.5		
Fully Connected Layer (200), Dropout = 0.5		
Softmax (100)		
